# When the Disperse Phase Crystallizes: How Surfactant Structure Shapes Interfacial Properties

**DOI:** 10.1021/acs.langmuir.5c06380

**Published:** 2026-02-26

**Authors:** Kerstin Risse, Stephan Drusch

**Affiliations:** Faculty III Process Sciences, Institute of Food Technology and Food Chemistry, Department of Food Technology and Food Material Science, Technische Universität Berlin, Straße des 17. Juni 135, Berlin 10623, Germany

## Abstract

Commercial oil–water emulsions typically contain a partially crystalline fat phase, which is essential for macroscopic attributes such as creaminess and whippability. While it is well established that the molecular structure of surfactants can accelerate or delay fat crystallization, much less attention has been paid to what happens at the interface during this process. Particularly, the extent to which fat crystallization modifies interfacial rheological properties remains insufficiently understood, despite their relevance for emulsion stability and functionality. This study investigates how cooling-induced crystallization of triglycerides affects interfacial viscoelasticity as a function of surfactant. Surfactants with identical saturated fatty acyl (FA) chains (C18:0) but different headgroups (Tween 60, BrijS20, Span 60), as well as Tweens with varying FA chain lengths (Tween 20: C12:0; Tween 60: C18:0), were examined. To capture differences in molecular similarity, tristearin (TS), tripalmitin (TP), and trilaurin (TL) in MCT oil were used as the fat phase. C18:0-based surfactants promoted interfacial TS crystallization and formed crystalline interfacial networks with increased viscoelasticity. Span 60 generated the strongest elastic response due to its dense interfacial packing and formation of a crystalline emulsifier layer, whereas Tween 60 and BrijS20 produced weaker, less connected structures. The FA chain length controlled the packing density and mobility of the interfacial (sub)­layer and thereby the resulting interfacial viscoelasticity upon cooling. Tween 20 formed a thin and highly mobile interfacial layer that disrupted TS and TP crystallization, resulting in weaker and less connected interfacial films compared to Tween 60 (C18:0). Overall, the results show that crystallization of the dispersed fat phase actively reshapes the structure, thickness, and connectivity of the interfacial layer, thereby altering interfacial viscoelasticity. The magnitude of this effect depends on the surfactant headgroup, FA chain length, and their molecular match with the triglyceride phase, which collectively determines the extent to which interfacial networks can form.

## Graphical abstract

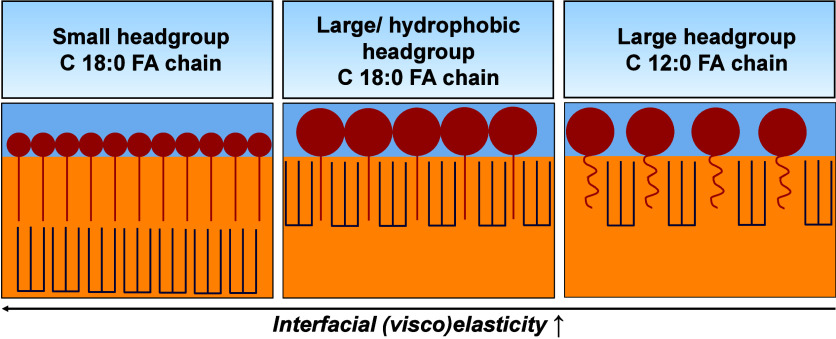

## 1 Introduction

Commercial oil–water emulsions often contain a certain amount of solid fat, which is responsible, among other things, for the creamy mouthfeel and whippability of the product. For instance, in dairy products, milk fat is partially crystalline from approximately −40 to +40 °C. Under refrigerated storage conditions (4–7 °C), the solid fat content is 50–70%, at room temperature around 20%, and during consumption and digestion (37 °C), it ranges from 0.3 to 5%.^1,2^

In industrial food processing, emulsification is generally carried out at temperatures at which the lipid phase is fully molten. The process can be divided into three key steps. First, both the aqueous and oil phases are heated to temperatures at or above the melting point to obtain completely liquefied triglycerides and, where needed, to dissolve the emulsifier. For low molecular weight emulsifiers (LME), this heating step may additionally induce a phase transition of the fatty acid chains (FA chains) toward a more fluid, disordered state. In the second step, the molten lipid phase is dispersed into the aqueous phase, generating fresh interface onto which the solubilized emulsifier can rapidly adsorb, forming a thin interfacial film around the droplets. Finally, the emulsion is cooled to ambient or product-specific temperatures. Upon cooling, the dispersed lipid phase may (at least partly) crystallize. Depending on the LME structure and the melting point of its FA chains, the emulsifier itself may undergo temperature-dependent phase transitions.^3,4^

It is generally accepted that the molecular structure of the LME affects the crystallization of the dispersed phase during cooling. Depending on the type of LME’s FA and their similarity to the FAs of the disperse phase, the LME may accelerate or decelerate the crystallization of the disperse phase.^5–4^ If the FA chain of the LME has a higher crystallization point than the emulsified triglycerides (Tm, LME > Tm, triglycerides), the high melting LME may function as a template for heterogeneous crystallization upon the cooling step, accelerating the crystallization of the disperse phase.^7–9^ When the FA chain of the LME has a lower melting point than the oil phase, on the other hand, the LME acts as an impurity in the crystallization of the dispersed phase. The result is the formation of less perfect crystals and a loosely packed lattice.^10,11^

Although the influence of LME molecular structure on the crystallization of emulsified fat is well established, the effects of the crystallization of the disperse phase on interfacial rheological properties remain less well characterized. In a previous study, we demonstrated that both the LME headgroup and the fatty acid (FA) chain length and saturation significantly influence interfacial behavior and the resulting interfacial rheological properties.^12–14^ For instance, we have shown that saturated PLs can form interfacial networks due to chain crystallization of the PL’s FA during cooling, increasing interfacial viscoelasticity.^13,14^ Others have linked this to molecular ordering or enhanced interactions among the headgroups of lipid-based emulsifiers at the interface.^14,15^ Unsaturated PLs, on the other hand, do not crystallize at the interface due to the lower chain melting point and the bent in the molecule that hinders the PLs from packing tightly at the interface.^13,14^ A similar trend was observed for nonionic LMEs such as for different Tweens vs Spans.^12^ Clearly, the LME’s molecular structure has an impact on 1) the interfacial packing 2) LME-LME interactions and 3) the crystallization of the dispersed phase. Melting and crystallization events at the interface may markedly affect interfacial viscoelastic properties.^11,16^ Yet, the complex interplay between these aspects remains to be understood.

The aim of this study was, therefore, to analyze how the crystallization of the disperse phase upon cooling affects the interfacial rheological properties as a function of the LME’s molecular structure. Tween 60 (large, strongly hydrophilic ethoxylated sorbitan headgroup), BrijS20 (large, rather hydrophobic polyethene glycol headgroup) and Span 60 (small, rather small sorbitan headgroup), each with a saturated C18:0 FA chain, were used to analyze the influence of the headgroup on the resulting interfacial structures. In a second step, we examined the influence of FA chain length by comparing two LMEs with the same headgroup (polyoxyethylene sorbitan) but different chain lengths, namely Tween 20 (C12:0) and Tween 60 (C18:0). To account for the molecular similarity between LME FA chains and the dispersed lipid phase, different triglyceride systems were employed: MCT oil with tristearin (TS), trilaurin (TL), and tripalmitin (TP).

## 2 Materials and Methods

TWEEN 20 (CAS 9005-64-5), TWEEN 60 (CAS 9005-67-8), and SPAN 60 (CAS S7192421) were purchased from Carl Roth GmbH (Karlsruhe, Germany). Brij S20 (Polyoxyethylene (20) stearyl ether) was purchased from Sigma-Aldrich (St. Louis, MO, USA). Medium chain triglyceride oil (MCT-oil) WITARIX MCT 60/40 was kindly provided by IOI Oleo GmbH (Hamburg, Germany). Sigma-Aldrich (St. Louis, MO, USA). Additionally, tristearin, trilaurin, and tripalmitin, purchased from Sigma-Aldrich (St. Louis, MO, USA), were used.

### 2.1 Sample Preparation

The Tweens and BrijS20 were dissolved in distilled water (0.01 wt % each), while the Span was dissolved in MCT oil (0.2 wt %). The choice of concentration was based on a previous study and corresponded to the critical micelle concentration.^12^ All four LMEs are nonionic, meaning their headgroups remain uncharged across different pH values. To ensure comparability, the pH of the aqueous phase was adjusted to approximately 6.

Both the aqueous and oil LME solutions were heated to 55 °C for 30 min before the measurements to melt the fatty acyl chains fully and to eliminate potential crystalline memory effects (i.e., reorganization into a previously ordered state upon cooling).^17^ The heat treatment protocol was based on differential scanning calorimetry (DSC) measurements.

Tripalmitin (TP), tristearin (TS), and trilaurin (TL) were each dissolved in medium-chain triglyceride (MCT) oil at a concentration of 10% (w/w). The mixtures were heated for 30 min at a temperature 10 °C above the melting points of the respective triglycerides (TP: 67.4 °C, TS: 72.5 °C, TL: 46.5 °C; based on DSC measurements) to ensure complete dissolution. The oil phases were prepared on a temperature-controlled magnetic heating plate placed directly next to the rheometer. Subsequently, the hot solutions were used directly for sample preparation without cooling.

### 2.2 Interfacial Shear Rheology

The interfacial shear rheology of Tween 20, Tween 60, BrijS20, and Span 60 was performed using an MCR 301 rheometer (Anton Paar GmbH, Ostfildern, Germany) with RheoCompass Software v1.25. A mini IRS measuring cell (d = 80 mm, height = 28.2 mm) and a biconical measuring geometry (Bic68-5; diameter 68.183 mm; α = 4.983°) were used.

40 mL of the aqueous phase was filled into the temperature-controlled and thermally insulated measuring cell, ensuring no air bubbles entered. The measuring cell itself was maintained at 20 °C. The bicone was positioned at the oil–water interface (T 20 °C), and 40 mL of the hot oil phase (preheated above the melting points of the triglycerides; TP: 67.4 °C; TS: 72.5 °C; TL: 46.5 °C), was carefully poured on top while still hot and the measurement was started without delay. The crystallization of the dispersed triglycerides thus occurred only after or during the transfer into the measuring system.

Measurements were conducted at 20 °C in three steps: time sweep (1% strain, 0.3 or 0.01 Hz, 1 h), frequency sweep (0.001–1 Hz, 0.1% strain), and amplitude sweep (0.1–100% strain, 0.3 or 0.01 Hz). Because a direct temperature sweep was not technically feasible, the initial time-sweep measurement simultaneously served as a controlled cooling and crystallization step. Since the same oil volume, transfer protocol, initial temperature (aqueous phase: 20 °C, oil phase: *T* > *T*_m_) and time sequence were used for all experiments, comparable cooling rates and reproducible crystallization conditions were ensured across all systems. During this initial period, the density and viscosity of the oil phases evolve significantly as a function of temperature and crystallization. For this reason, the time-sweep was used solely to monitor the formation and stabilization of the interfacial layer and was not subjected to quantitative analysis. All subsequent rheological measurements and data evaluation were performed exclusively at the stabilized temperature of 20 °C, using the corresponding density and viscosity parameters at this temperature. Here, G_i_′ and G_i_″ were calculated from the phase shift between deformation and shear stress and plotted as a function of strain (amplitude sweep).

Oscillatory deformation was applied via the rotating bicone. Storage (G′) and loss (G″) moduli were plotted against strain. The linear viscoelastic (LVE) limit was defined as the strain at which G′ deviated by 3% from its initial value. Additionally, Lissajous plots were used to characterize the interfacial behavior in the LVE and nonlinear (NLVE) regimes. In these plots, perfect circles indicate ideal viscous behavior, while straight lines represent ideal elastic behavior (and vice versa, depending on the type of plot).

To further analyze interface behavior, the stiffening factor S and the thickening factor T were calculated according to^18^ as follows:

$$S=\frac{{G^{\prime }}_{L}-{G^{\prime }}_{M}}{{G^{\prime }}_{L}}$$ 1

and

$$T=\frac{{\eta^{\prime }}_{L}-{\eta^{\prime }}_{M}}{{\eta^{\prime }}_{L}}$$ 2

where G′_L_ and η′_L_ refer to values at maximum strain, and G′_M_ and η′_M_ to those at minimum strain. S ≈ 0, T ≈ 0 indicates linear viscoelastic behavior. S < 0 indicates strain-softening. S > 0 indicates strain-hardening. *T* < 0 indicates intracycle shear thinning. T > 0 indicates intracycle shear thickening.

To test if the crystallization of the dispersed phase has an impact on the measurements the Boussinesq number was calculated. In interfacial shear rheology, the contribution of the interfacial layer to the measured mechanical response is often evaluated using the Boussinesq number (Bo).

The Boussinesq number describes the ratio between interfacial viscoelastic forces and bulk viscous forces and is calculated according to the following equation:

$$Bo=\frac{surface\;drag}{subphase\;drag}=\frac{G_{i}^{\prime }}{\eta \omega }$$ 3

where G′_i_ is the interfacial storage modulus (Pa·m), η is the dynamic viscosity of the bulk phase (Pa·s), and ω is the angular frequency (rad/s) (here f = 0.01 Hz, ω=2πf = 0.0628 rad/s).

A Boussinesq number significantly greater than 1 indicates that the interfacial properties dominate the mechanical behavior, while values much smaller than 1 suggest that bulk viscosity effects prevail.^19^

Based on that, the solid fat content within the oil phase was limited to 10% (w/w) to only measure the viscoelasticity of the interface without capturing any bulk-dominated effects (Bo ≫ 1). The corresponding Boussinesq numbers for all experimental conditions are provided in Section 3.1.

All experiments were performed in triplicate. Mean values and standard deviations of G′, G″, S, and T were calculated. One representative Lissajous plot per condition was selected for illustration.

## 3 Results and Discussion

### 3.1 Molecular and Interfacial Characteristics of the Surfactants

To support the interpretation of the following results, we first estimated the molecular packing characteristics of the different surfactants at the oil–water interface (Table 1). All parameters were determined at a water-liquid MCT interface, i.e. under conditions where no crystallization of the dispersed phase occurs, and thus serve as a reference framework for the subsequent analysis. The CMC, equilibrium interfacial tension and molecular area values used here were taken from our previous publication. Building on this data set, we additionally derived further interfacial and packing parameters in the present work. For experimental details and exact values of the primary interfacial properties, the reader is referred to the earlier publication.^12^

**Table 1:** Interfacial Properties and Derived Packing Parameters of Tween 20, Tween 60, Span 60, and BrijS20 Critical Micelle Concentration (CMC), Equilibrium Interfacial Tension (IFT), Gibbs Adsorption Isotherm Γ, Molecular Area A, Estimated Lateral Spacing d, and Packing Factor P^c^

Headgroup	FA chain	Surfactant	IFT at CMC [mN/m]	CMC [wt %]	CMC x 10–6 [mol/]	**Γ** [μmol/m2]	A [nm^2^]	d [nm]^a^	P [nm^3^]^b^
Ethoxylated sorbitan	C 12:0	Tween 20	5.2	0.01	9.22	0.47	3.55	2.00	0.05
	C 18:0	Tween 60	7.6	0.01	7.07	0.34	4.92	2.24	0.04
Sorbitan		Span 60	10.2	0.25	591.67	1.10	1.52	1.41	0.11
Glycol		BrijS20	6.3	0.01	5.22	0.26	6.56	2.65	0.03

a *Here the characteristic lateral spacing d between neighboring molecules was estimated from the molecular area A as* $d\approx \sqrt{A}$ . *This follows directly from simple geometric considerations: in a two-dimensional interfacial layer, each molecule occupies on average an area A, and the corresponding characteristic intermolecular distance is therefore given by the square root of this area.*

b *Here we calculated P according to Schwenke* *& Tanford (1973)* ^20^ *with P =* $\frac{v}{a_{0}l}$ *with v = hydrophobic chain volume = 0.1265 n + 0.15 nm, l max. chain length = 0.0269n + 0.0274, a* **_0_** *= Area per molecule.*

c *Please note that the CMC, IFT,* Γ *and A are part of our previous study (Risse & Drusch, 2024).*

Clear quantitative differences are observed between the molecular packing parameters of the investigated surfactants (Table 1). Tween 20 and Tween 60 exhibit low equilibrium interfacial tensions at the CMC (5.2 and 7.6 mN m^–1^, respectively) and relatively low surface excess values derived from the Gibbs adsorption isotherm (Γ = 0.47 and 0.34 μmol m^–2^), corresponding to larger molecular areas of 3.55 and 4.92 nm^2^ and larger estimated lateral spacings d. In contrast, Span 60 shows a substantially higher surface excess (Γ = 1.10 μmol m^–2^), a markedly smaller molecular area (1.52 nm^2^), reduced lateral spacing d, and a correspondingly higher packing factor P, indicative of much denser interfacial packing. Brij S20 displays intermediate characteristics, with Γ = 0.26 μmol m^–2^, a comparatively large molecular area of 6.56 nm^2^, and lower packing factor P.

These quantitative differences reflect systematic variations in interfacial organization arising from both headgroup chemistry and fatty acid chain length. Despite their similar overall molecular architecture, relatively small variations in headgroup chemistry or fatty acid chain length are sufficient to induce pronounced changes in interfacial packing and composition, as reflected by differences in Γ, d, and P. Ethoxylated surfactants (Tween 20 and Tween 60) form comparatively loose, sterically stabilized interfacial layers, whereas Span 60 forms a densely packed interfacial film. Brij S20 exhibits behavior intermediate between these two extremes.

### 3.2 Critical Assessment of Measurement Regime

In the next step, we moved on to interfacial shear rheology under conditions where crystallization of the dispersed phase can occur, using different triglyceride mixtures (TL: Trilaurin, TP: Tripalmitin, TS: tristearin). This marks the central focus of the present study, as interfacial rheology is here used to directly probe the coupling between fat crystallization and interfacial mechanical response.

As the presence of crystalline fat in the dispersed phase may potentially influence the measured response, we subsequently performed a critical assessment of the reliability and origin of the rheological signal. In particular, an increasing contribution of the oil phase could, in principle, affect the measured moduli. To address this point, we evaluated the measurement regime using the Boussinesq number and analyzed its dependence on deformation amplitude (Figures 1 and 2; see also Materials and Methods). This approach allows us to assess whether the obtained response is dominated by interfacial contributions or increasingly influenced by bulk effects.

**Figure 1:**
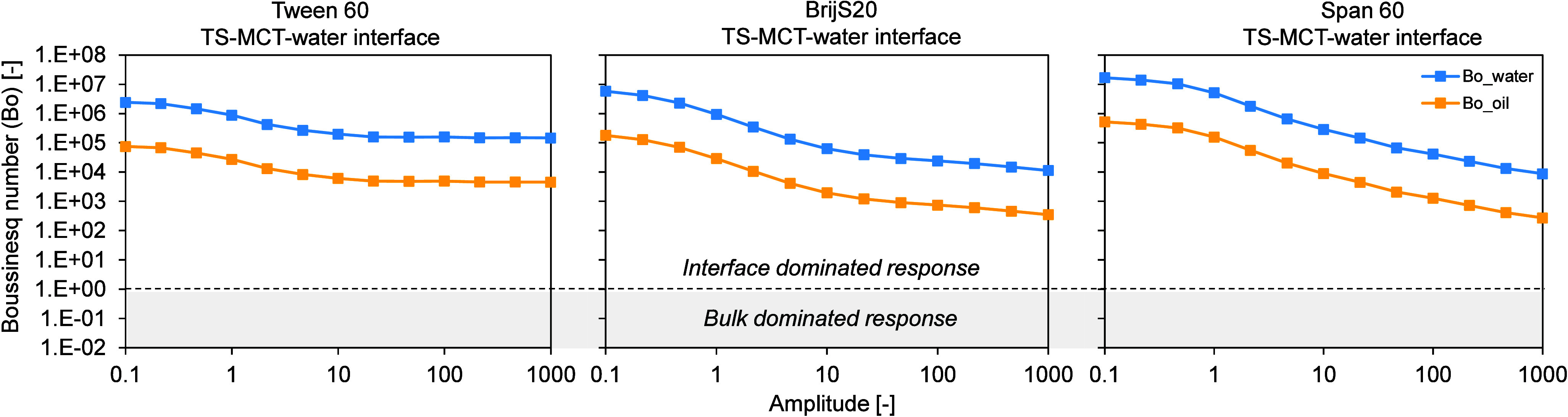
Boussinesq number as a function of deformation amplitude for aged emulsifier films at the TS-MCT–water interface. The Boussinesq number was calculated from interfacial shear rheology data obtained during amplitude sweeps at a constant frequency of 0.01 Hz over an amplitude range of 0.1–100 (−), measured at T = 20 °C after cooling the oil phase to 20 °C. Measurements were performed at the respective CMC of each low-molecular-weight emulsifier (LME): Tween 60 (0.01 wt %), Brij S20 (0.01 wt %) and Span 60 (0.2 wt %). Panels show results for Tween 60 (left), Brij S20 (center) and Span 60 (right). TS denotes tristearin. The amplitude sweep is shown in double-logarithmic representation.

**Figure 2:**
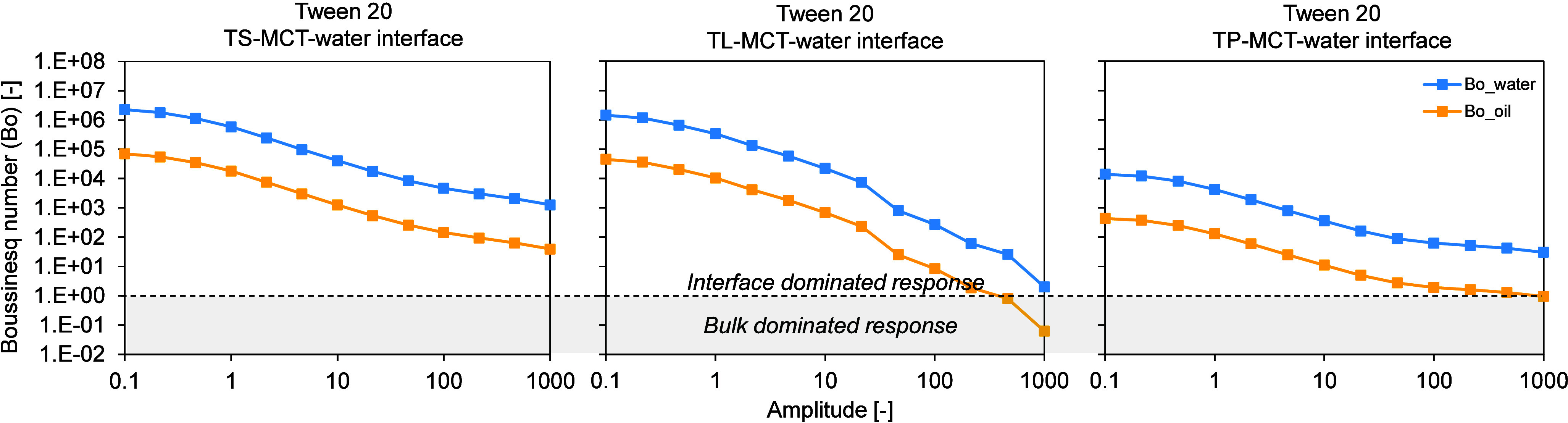
Stress response of the interfacial layer formed by an aged Tween 20 emulsifier film at the TS-MCT–water (left), TL-MCT–water (center), and TP-MCT–water (right) interfaces, determined by interfacial shear rheology. Amplitude sweeps were performed at a constant frequency of 0.01 Hz over an amplitude range of 0.1–100 (−) at T = 20 °C, after cooling the oil phase to 20 °C. Interfacial rheological measurements were conducted at the CMC of the low-molecular-weight emulsifier Tween 20 (0.01 wt %). TS, TL and TP denote tristearin, trilaurin and tripalmitin, respectively. The amplitude sweep is shown in double-logarithmic representation.

For all investigated systems, including both the Tween 60-BrijS20-Span 60 series and the Tween 20 systems, the calculated Boussinesq numbers are well above one, with values on the order of 10^7^–10^8^ at low deformation amplitudes (beginning of the amplitude sweep) (Figures 1 and 2). When evaluated with respect to the aqueous phase, Boussinesq numbers reach values up to 10^7^–10^8^ at the beginning of the amplitude sweep, whereas values calculated using the oil phase as reference are systematically lower, on the order of 10^5^, suggesting that the contribution of the oil phase to the measured signal is more pronounced than that of the aqueous phase. Nevertheless, in both cases the measured rheological response is clearly dominated by interfacial contributions at low amplitudes, and the impact of the bulk phase on the interfacial response is negligibly small.

With increasing deformation amplitude, the Boussinesq number gradually decreases for all systems, reflecting an increasing contribution of bulk dissipation at larger deformations. While the response remains interface-dominated over a wide amplitude range, a more pronounced decrease is observed for the Tween 20 systems, where the Boussinesq number approaches values close to one at amplitudes above 100 [−].

Based on this observation, the interpretation of the interfacial rheological data was therefore restricted to deformation amplitudes ≤100 [−], where interfacial dominance can be ensured for all systems. Although Tween 60, Brij S20 and Span 60 remain well within the interface-dominated regime even at higher amplitudes, a uniform amplitude range was chosen for consistency across all surfactants.

### 3.3 Interfacial Rheology of Surfactant-Stabilized TAG-MCT–Water Interfaces

We next proceed to the interpretation of the interfacial shear rheology results obtained for surfactant-stabilized water–MCT interfaces in the presence of crystallizing triglycerides. Here, the focus is placed on how the interfacial mechanical properties evolve when crystallization occurs in the dispersed phase, and how this response depends on surfactant molecular structure. In the following, we first address the influence of the surfactant headgroup by comparing Tween, Brij, and Span systems, and subsequently examine the effect of fatty acid chain length using Tween 20 and Tween 60.

Figure 3–5 shows the results of amplitude sweep measurements for oil–water interfaces stabilized with Tween 60, BrijS20, and Span 60. The oil phase consisted of tristearin (TS) mixed with MCT oil, and the amplitude sweeps were conducted at a constant frequency of 0.01 Hz and a temperature of 20 °C. The oil phase was added to the system while it was still hot (>50 °C) and was allowed to cool to 20 °C during the time sweep (i.e., prior to the amplitude sweep), leading to crystallization at the interface.

**Figure 3:**
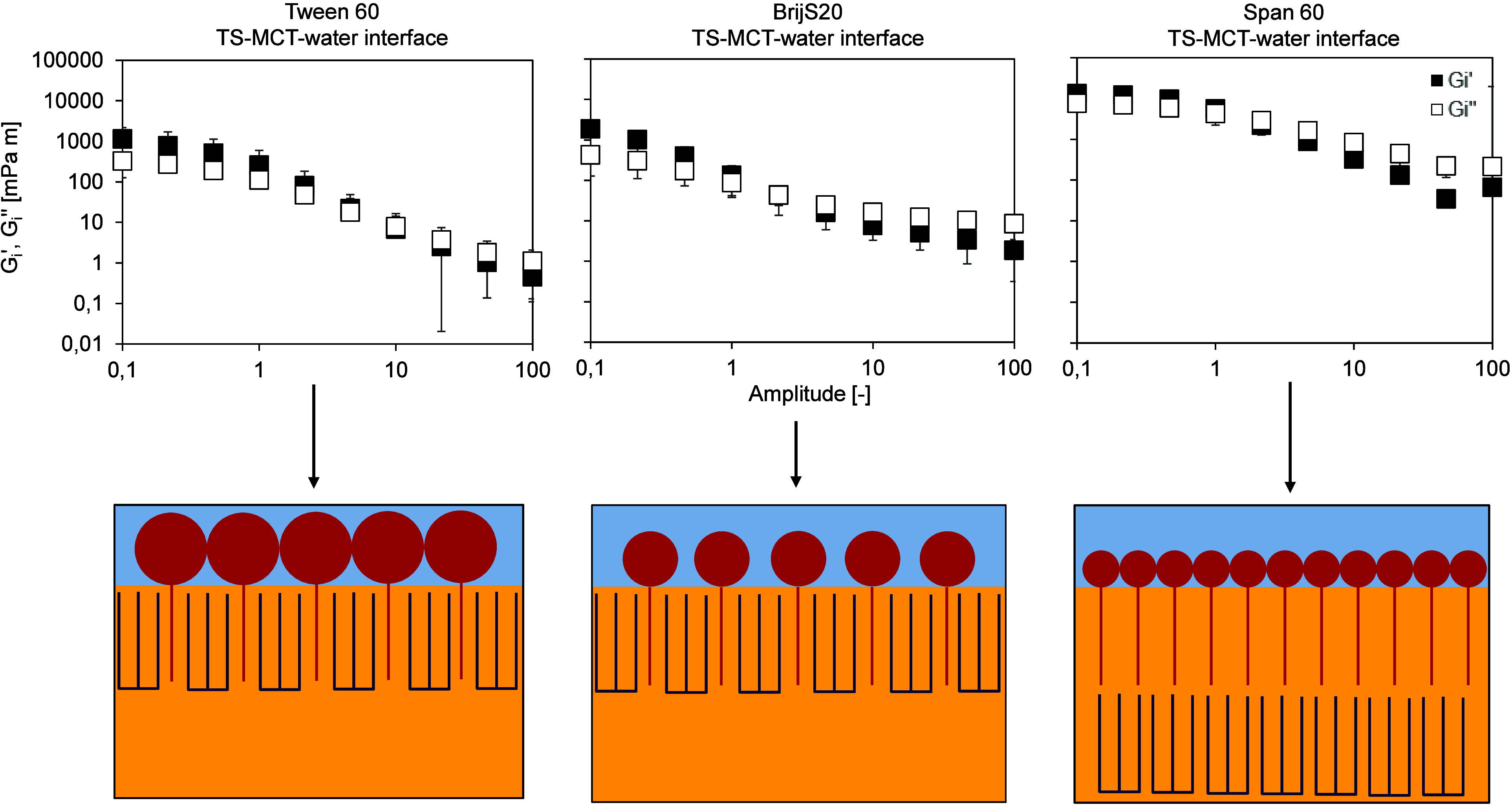
Stress response of the interfacial layer of the aged emulsifier film at the TS-MCT–water interface, determined by interfacial shear rheology (amplitude sweep at a constant frequency of 0.01 Hz, amplitude range 0.1–100 [−]), performed at T = 20 °C after cooling the oil phase down to 20 °C. Interfacial rheological properties were determined at the LME’s CMC: Tween 60:0.01 wt %, BrijS20:0.01 wt % and Span 60:0.2 wt %, respectively. Left: Tween 60. Centre: BrijS20. Right: Span 60. TS: tristearin. The amplitude sweep is shown in double-logarithmic representation.

In all cases, the storage modulus G_i_′ was larger than the loss modulus G_i_″ (Figure 3), indicating a viscoelastic network. This is shown in the Lissajous plots by a predominantly elastic response (small ellipsoidal curves with rather large slopes, Figure 4). As a comparison, at the MCT-oil–water interface (i.e., without TS), Tween 60 and BrijS20 interfaces behaved predominantly viscous and only Span 60 formed a viscoelastic network at the interface with the storage moduli values being significantly lower than at the TS-MCT–water interface (see ref 12 ) Apparently, all three emulsifiers promoted the formation of a crystalline tristearin (TS) network at the interface through a templating effect, as confirmed by the high storage modulus values. The interfacial storage modulus of Span 60 at the TS-MCT–water interface is approximately 1 order of magnitude higher (≈10000 mPa·m) than that of Tween 60 and Brij S20 (≈ 1000 mPa·m), indicating a significantly stiffer and more elastic interfacial network for Span 60. Due to the lower interfacial coverage (lower CMC) in the case of Tween 60 and BrijS20 (see Table 1, Section 3.1), the TS networks formed with these emulsifiers were less compact and, therefore, less stable compared to the TS network formed by Span 60. A densely packed interface likely facilitates the formation of a compact crystalline network at the fluid–fluid interface, where the surfactant plays a templating role in heterogeneous crystallization. In an emulsion system, where the interface is curved and the oil phase is present as discrete droplets, this implies that crystallization of the dispersed phase is largely restricted to the droplet interior, as the (crystalline) interfacial layer acts as a barrier. Here, crystallization of TAG is likely to occur beneath the interfacial layer, leading to the formation of a crystalline sublayer. As a result, a compact and comparatively thick interfacial structure develops, comprising both the surfactant-rich layer and the underlying crystalline TAG sublayer, which together form a mechanically robust and elastic interfacial network (Figure 3, graphical illustration Span 60).

**Figure 4:**
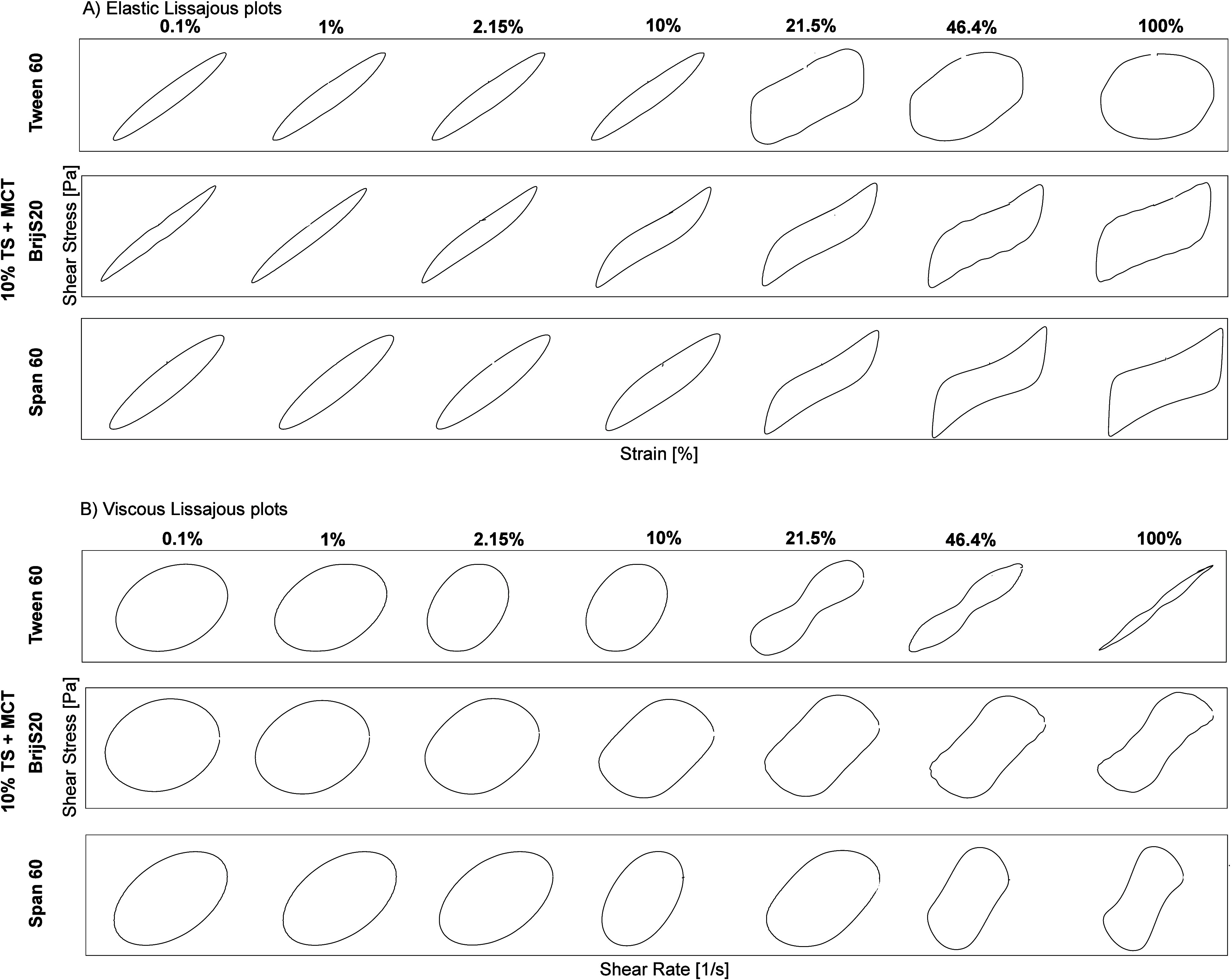
Stressing of the aged emulsifier film at the TS-MCT–water interface at T = 20 °C, determined by interfacial shear rheology using the rheometer. Lissajous plots plotted as strain plots for amplitudes of 0.1%, 1%, 2.15%, 10%, 21.5%, 46.4% and 100% to analyze the elasticity of the Tween 60, Span 60 and BrijS20 films at a frequency of 0.01 Hz (bottom row). Left: Tween 60. Centre: BrijS20. Right: Span 60. Interfacial rheological properties were determined at the LME’s CMC: Tween 60:0.01 wt %, BrijS20:0.01 wt % and Span 60:0.2 wt %, respectively. TS: tristearin. Lissajous plots are shown on linear axes.

In contrast, at lower interfacial coverage, as observed for Tween 60 and Brij S20, insufficient surfactant packing may allow growing fat crystals to locally disrupt or penetrate the interfacial film, leading to reduced interfacial integrity and increased susceptibility to interfacial instabilities. Here, TAG crystallization is more likely to occur between individual surfactant molecules rather than as a distinct sublayer. While this process can increase the apparent interfacial thickness, it does not result in the formation of a continuous crystalline sublayer (Figure 3, graphical illustration Tween 60 & BrijS20). Consequently, the resulting interfacial structure remains overall thinner and mechanically weaker, which is reflected in lower interfacial storage moduli. This behavior is consistent with previous reports by Helgason et al. (2009), that showed that the crystallization behavior is governed by the degree of interfacial coverage. In this context, insufficient interfacial coverage may allow growing crystals to extend beyond the droplet interior and interact with the surrounding aqueous phase. Conversely, dense surfactant packing can lead to the formation of a rigid interfacial shell, which acts as a physical barrier and may promote templated crystallization confined to the droplet interior.^21^ In a similar manner, Fredrick et al. (2013) demonstrated that the chemical nature of the surfactant further impacts bulk fat crystallization, beyond effects related to interfacial coverage alone. For instance, saturated monoacylglycerols form a solid-like crystalline interfacial layer that promotes heterogeneous nucleation and suppresses crystal penetration through the interface, whereas unsaturated, liquid emulsifiers favor crystallization in the droplet interior and facilitate interfacial piercing.^22^

After the critical strain (1%), the Lissajous plots of all three interfaces become highly nonlinear, and the area between the curves increases, meaning that the interface becomes more viscous (Figure 4). In the case of the Tween 60 interface, we observe strain thinning (G_i_′, G_i_″ decreasing), and the area within the Lissajous plots become wider and wider until the Lissajous plot is nearly a perfect cycle at 100% deformation, suggesting that the interfacial structure broke down completely at this point and the deformation behavior is nearly fully viscous (Figures 3 and 4). Tween 60 had the largest headgroup of all, implying that the formed interfacial structures were the most loosely packed and were, therefore, more prone to break down during deformation.

In contrast, the Lissajous plots of the Span 60 and BrijS20 interfaces become increasingly pinched, forming narrow, constricted regions that indicate stiffening behavior.

To quantitatively study the Lissajous plots, we have determined the nonlinearity parameters S (stiffening factor) and T (thickening factor), as shown in Figure 5.

**Figure 5:**
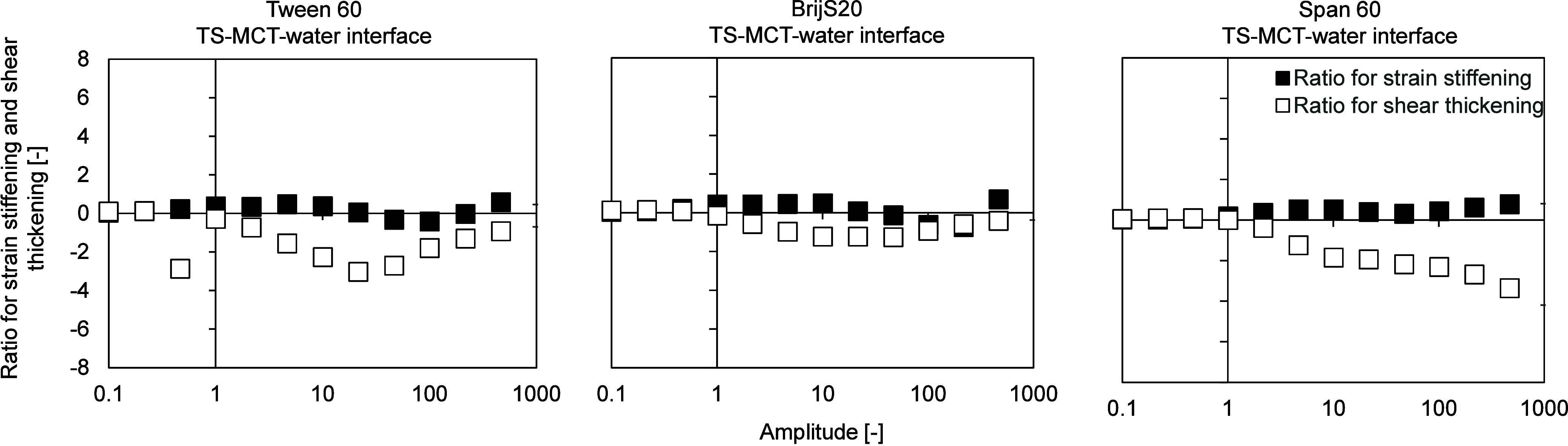
Ratio for strain stiffening and shear thickening of the aged emulsifier film at the TS-MCT–water interface at T = 20 °C, determined by interfacial shear rheology using the rheometer. Interfacial rheological properties were determined at the LME’s CMC: Tween 60:0.01 wt %, BrijS20:0.01 wt % and Span 60:0.2 wt %, respectively. Left: Tween 60. Centre: BrijS20. Right: Span 60. TS: tristearin. The *y*-axis is linear, the *x*-axis is plotted on a logarithmic scale.

For amplitudes lower than 1%, the S-factor and T-factor are both around 0 for all three interfaces, which indicates linear viscoelastic behavior (Figure 5). This behavior was already seen in Figure 3, where we detected a linearity limit of 1%. After reaching the linearity limit (>1%), the T-factor increases slightly, then decreases and becomes negative, indicating pronounced shear thinning behavior, where the viscous response of the material weakens with increasing deformation. This was particularly the case with the Span 60 interface. The S-factor, on the other hand, remained low over the range of amplitudes, with the trend being toward strain stiffening. Still, the overall response seemed to be shear thinning.

To specifically investigate the influence of dispersed phase crystallinity on interfacial properties, Tween 20 was selected as the emulsifier. Due to its shorter hydrophobic chain (C12:0), Tween 20 forms a more flexible interfacial film compared to emulsifiers with longer saturated chains, such as Tween 60 (C18:0), without crystallizing itself.^12^ This minimized the potential impact of the emulsifier on the interfacial mechanical properties, allowing for a more precise assessment of the effects originating solely from the crystallization behavior of the dispersed triglycerides. Figures 6–8 show the results of amplitude sweep measurements for the matured Tween 20-stabilized interfacial layers at the TS-MCT–water, TL-MCT–water, and TP-MCT–water interfaces. The curves depict the evolution of the storage modulus (G_i_′) and loss modulus (G_i_″) as a function of strain amplitude at a constant frequency of 0.01 Hz and T = 20 °C.

**Figure 6:**
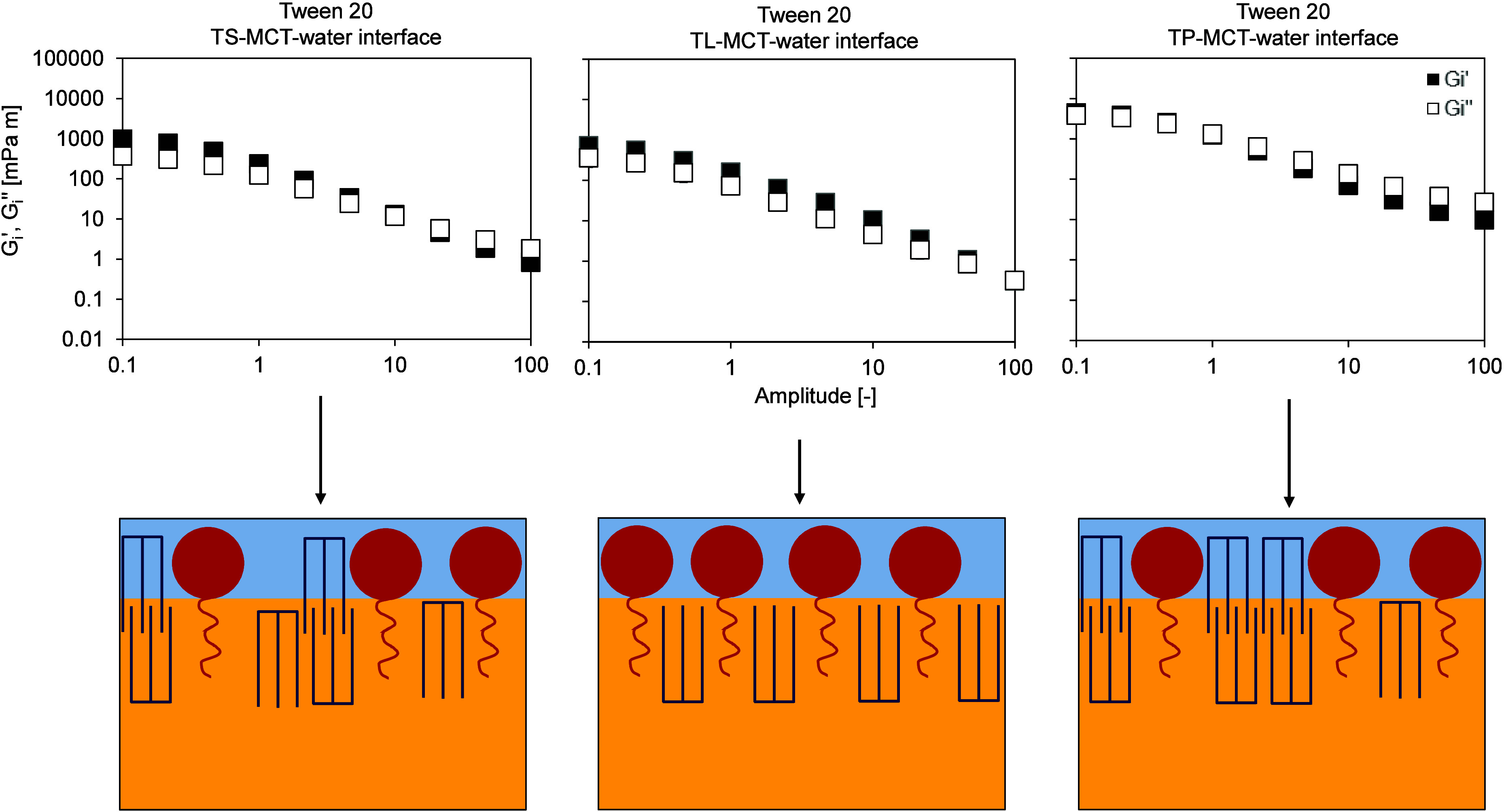
Stress response of the interfacial layer of the aged Tween 20 emulsifier film at the TS-MCT–water (left), TL-MCT–water (center), and TP-MCT–water (right) interfaces, determined by interfacial shear rheology (amplitude sweep at a constant frequency of 0.01 Hz, amplitude range 0.1–100 [−]), performed at T = 20 °C after cooling the oil phase down to 20 °C. Interfacial rheological properties were determined at the LME’s CMC: Tween 20:0.01 wt %. TS: tristearin; TL: trilaurin; TP: tripalmitin. The amplitude sweep is shown in double-logarithmic representation.

**Figure 7:**
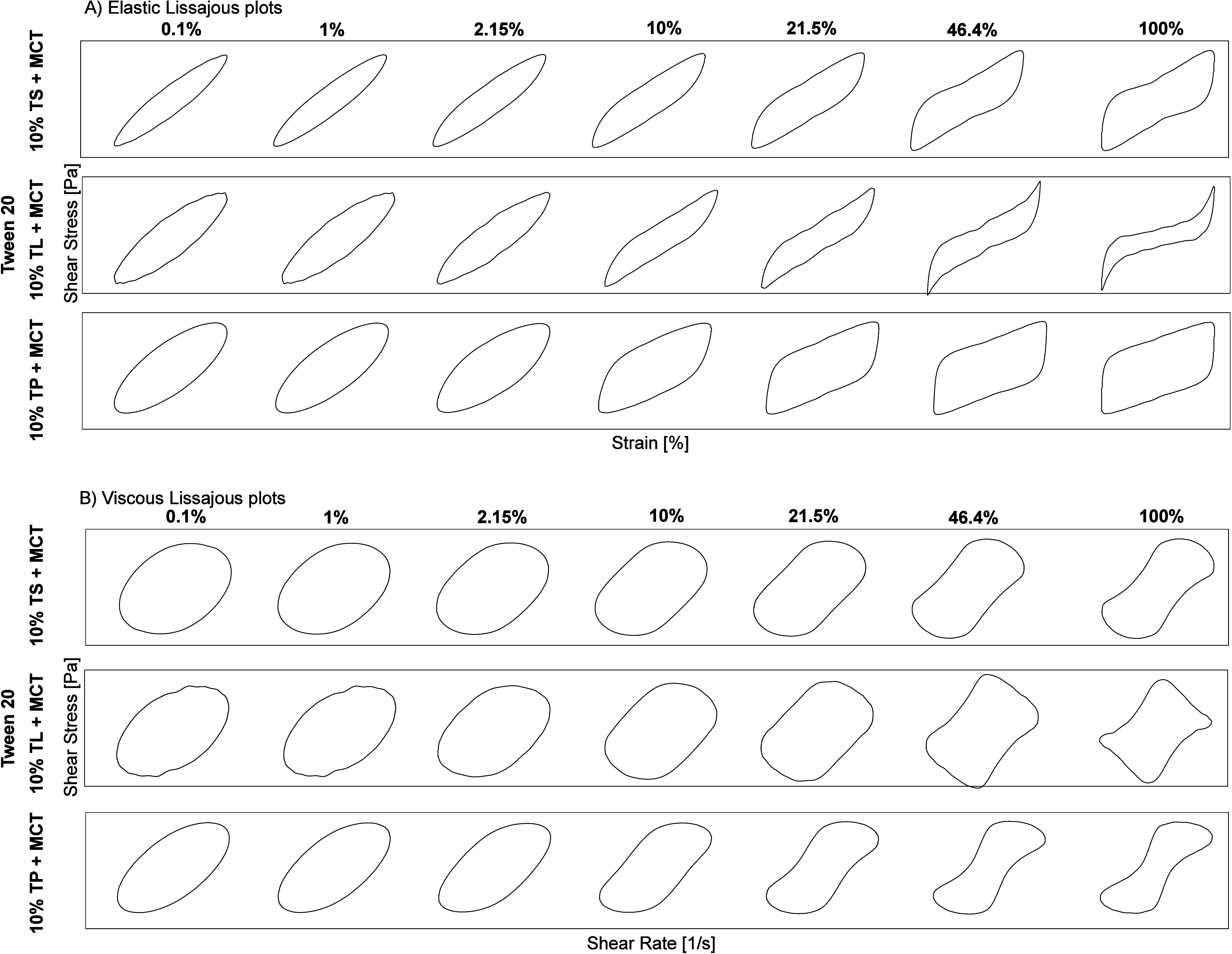
Stressing of the aged Tween 20 film at the TS-MCT–water (left), TL-MCT–water (center), and TP-MCT–water (right) interfaces, determined by interfacial shear rheology. Lissajous plots plotted as strain plots for amplitudes of 0.1%, 1%, 2.15%, 10%, 21.5%, 46.4% and 100% to analyze the elasticity of the Tween 60, Span 60 and BrijS20 films at a frequency of 0 0.01 Hz (bottom row). Interfacial rheological properties were determined at the LME’s CMC: Tween 20:0.01 wt %. TS: tristearin; TL: trilaurin; TP: tripalmitin. Lissajous plots are shown on linear axes.

**Figure 8:**
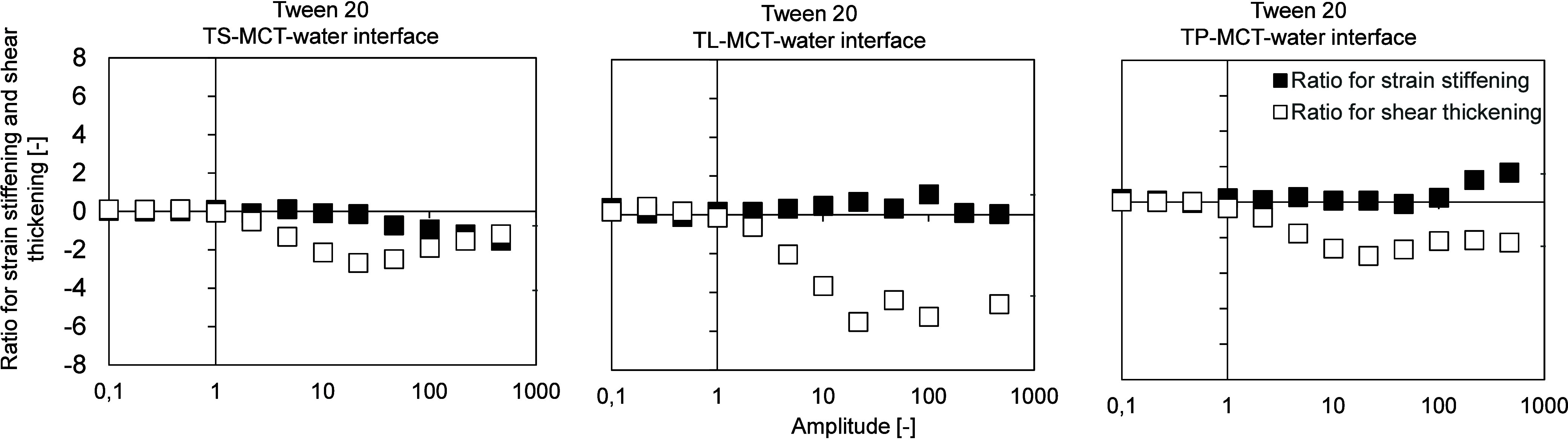
Stress response of the interfacial layer of the aged Tween 20 emulsifier film at the TS-MCT–water (left), TL-MCT–water (center), and TP-MCT–water (right) interfaces, determined by interfacial shear rheology (amplitude sweep at a constant frequency of 0.01 Hz, amplitude range 0.1–100 [−]), performed at T = 20 °C after cooling the oil phase down to 20 °C. Interfacial rheological properties were determined at the LME’s CMC: Tween 20:0.01 wt %. TS: tristearin; TL: trilaurin; TP: tripalmitin. The *y*-axis is linear, the *x*-axis is plotted on a logarithmic scale.

As shown in Figure 6, for all three Tween 20 interfaces (TS-MCT-water; TL-MCT-water; TP-MCT-water), the storage modulus G_i_′ was higher than the loss modulus G_i_″ at low amplitudes, indicating a viscoelastic deformation behavior. Both the storage and loss moduli showed a slight decrease even at low amplitudes above 5%, suggesting that the critical strain, which marks the limit of the linear viscoelastic (LVE) region, was already exceeded at amplitudes greater than 0.1%.

The TP-MCT oil–water interface stabilized with Tween 20 exhibited the highest values for both storage and loss moduli. However, the crossover point of storage and loss moduli occurred earlier compared to the other two interfaces (Figure 6). In the same way, the area within the Lissajous plots was the largest for this interface (Figure 7). After an amplitude of 2.15%, the loss modulus of the Tween 20-stabilized TP-MCT–water interface was higher than the storage modulus (Tween 20 TS: 9.99%, Tween 20 TL: 46.4%), and the T-value became negative, indicating the breakdown of crystalline structures, i.e., the onset of interfacial flow.

In contrast, at the TL-MCT oil–water interface stabilized with Tween 20, the crossover occurred at a much higher amplitude (46.5%, Figure 6). In the same way, the Lissajous plots showed pronounced strain stiffening, as indicated by the pinched, constricted regions at the top right corner of the curve (Figure 7). This behavior could be attributed to the different melting points of Tween 20 and the dispersed fats. The crystallization of TP and TS started at higher temperatures compared to TL (TP: melting point 44.7–67.4 °C; TS: 54–72.5 °C; TL: 46.5 °C). Likely, the FA chains of Tween 20 were still in a liquid state when the crystallization of TP and TS began. In fact, Tween 20 does not exhibit a well-defined solid–liquid phase transition and remains liquid at temperatures above 0 °C.^12^ Consequently, Tween 20 and TP or TS did not form a common crystalline network at the interface. Instead, the crystalline FA chains of Tween 20 acted as “impurities” within the crystalline TP or TS network, resulting in lower interfacial viscoelasticity. This is, among others, because during crystal growth, emulsifiers can adsorb at steps or kinks on the surface of growing fat crystals,^23^ thereby modifying crystal morphology and growth kinetics.^10,11,10^ Accordingly, Tween 20 layer does not form a sufficiently rigid or continuous barrier to confine crystallization beneath the interface. As a result, crystallization of both TP and TS was no longer confined to the droplet interior. Instead, growing fat crystals were observed to penetrate the Tween 20 interfacial film and extend into the continuous aqueous phase,^22^ as illustrated in Figure 6. At the TL-MCT oil–water interface stabilized with Tween 20, the crystallization of TL occurred at a lower temperature compared to TS or TP, allowing the simultaneous crystallization of TL and progressive ordering of Tween 20 at the interface, likely due to the close match between the melting characteristics of the dispersed fat phase and the FA chain of the surfactant (C 12:0 in both cases). This FA chain length compatibility facilitates a parallel reorganization of the Tween 20 interfacial layer and the crystallizing TL molecules, leading to the formation of an interconnected surfactant–TAG network (Figure 6, graphical illustration). As a result, emulsifier-TAG interactions are strengthened, yielding a more coherent interfacial structure compared to systems lacking such chain-length matching. Overall, this results in a more stable interfacial network compared to the other systems.

Finally, the viscoelastic behavior of Tween 20- and Tween 60-stabilized TS-MCT–water interfaces was compared to further investigate the influence of the emulsifier’s fatty acyl chain length (C12:0 for Tween 20 vs C18:0 for Tween 60) on interfacial properties, while keeping the headgroup constant (nonionic, polyoxyethylene-based). This approach enables a targeted assessment of how variations in the fatty acyl chain affect interfacial network formation and mechanical stability.

The storage modulus (G_i_′) of the Tween 60-stabilized interface was substantially higher across the entire strain range, indicating the formation of a more rigid and elastic interfacial network. In contrast, the interface stabilized by Tween 20 exhibited lower G_i_′ values and an earlier crossover between G_i_′ and G_i_″, suggesting a weaker and more deformable film (Figure 3 vs Figure 6). These differences can be attributed to the molecular structure of the emulsifiers: Tween 60, containing a longer hydrophobic stearic acid chain (C18:0), likely promotes stronger interactions and higher packing density at the interface compared to Tween 20, which is based on the shorter lauric acid chain (C12:0). Thus, the length of the FA chain in the LME plays a crucial role in determining the viscoelastic properties and structural stability of triglyceride-stabilized oil–water interfaces, which is in line with the literature. According to Garti & Sato (2001), increasing similarity in FA chain length and saturation between the LME and the emulsified triglycerides enhances their intermolecular interactions.^23^

In addition, Tween 60 exhibits a greater structural similarity to the dispersed phase, due to its C18:0 FA chain, compared to Tween 20, which contains a shorter C12:0 FA chain; this difference is expected to result in distinct effects on the crystallization behavior of the dispersed phase.^5–4^ If the FA chain of the LME has a higher crystallization point than the emulsified triglycerides (T_m, LME_ > T_m, triglycerides_) (which is the case for Tween 60), the high melting LME may function as a template for heterogeneous crystallization upon the cooling step, accelerating the crystallization of the disperse phase.^7–9^ When the FA chain of the LME has a lower melting point than the oil phase (which is the case for Tween 20), on the other hand, the LME acts as an impurity in the crystallization of the dispersed phase. The result is the formation of less perfect crystals and a loosely packed lattice.^10,11,10^ Tween 60 accelerated the crystallization of the disperse phase, which in turn led to stiffer interfaces. In contrast, Tween 20 was still in a liquid state at 20 °C and during cooling, Tween 20 was incorporated as impurities within the TS-crystals, and the resulting interfacial layer is less stiff than the Tween 60 interface.

For completeness, frequency sweeps were additionally performed (Figures 9 and 10). In all cases, an elastic-dominated response was observed, with Gi′ exceeding Gi″ over most of the investigated frequency range.

**Figure 9:**
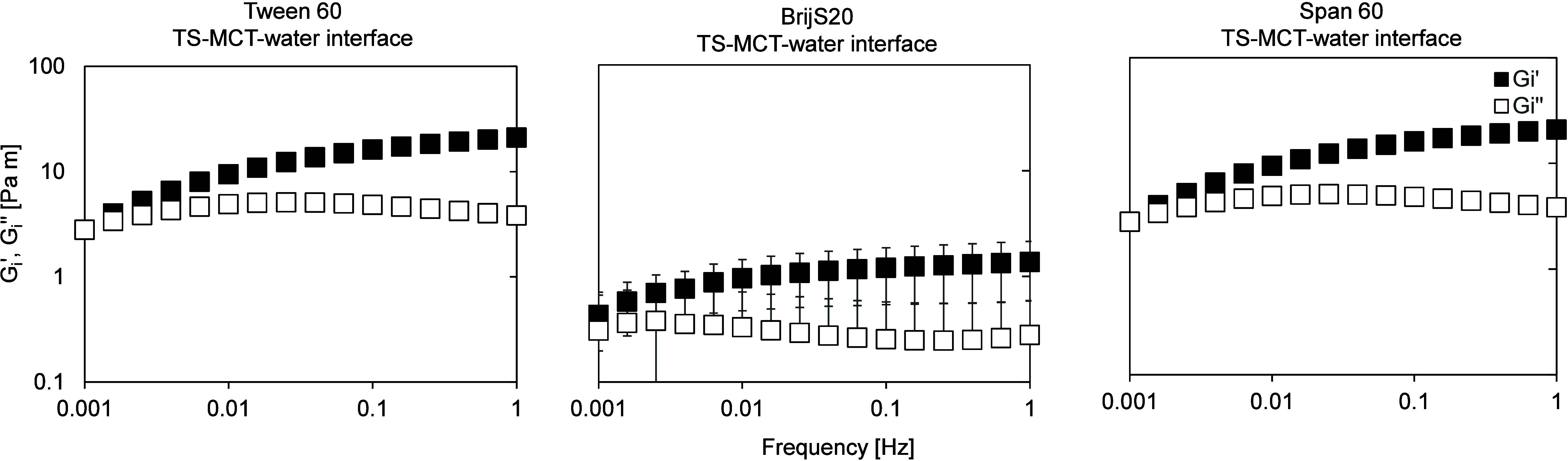
Frequency-dependent interfacial shear moduli of aged emulsifier films at the TS-MCT–water interface, determined by interfacial shear rheology. Frequency sweeps were performed at a constant strain amplitude of 0.1% over a frequency range of 0.001–1 Hz at T = 20 °C. Panels show results for Tween 60 (left), Brij S20 (center) and Span 60 (right). The frequency sweep is shown in double-logarithmic representation.

**Figure 10:**
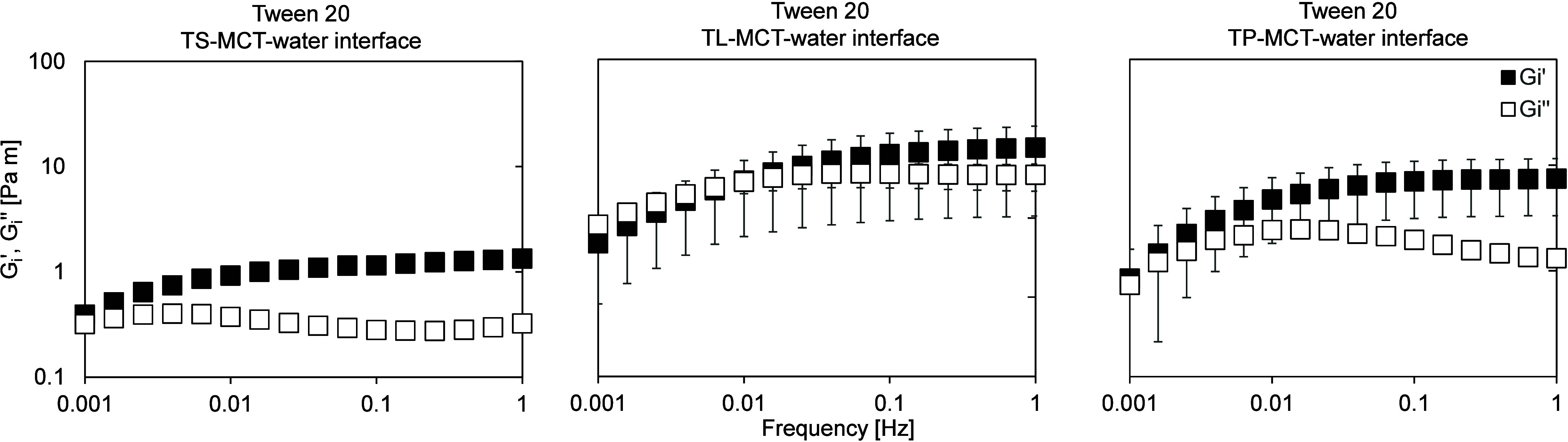
Frequency-dependent interfacial shear moduli of aged Tween 20 films at the TS-MCT–water (left), TL-MCT–water (center), and TP-MCT–water (right) interfaces, determined by interfacial shear rheology. Frequency sweeps were performed at a constant strain amplitude of 0.1% over a frequency range of 0.001–1 Hz at T = 20 °C. The frequency sweep is shown in double-logarithmic representation.

For all systems, Gi′ and Gi″ vary with frequency, indicating a frequency-dependent interfacial response that likely reflects a convolution of viscoelastic and time-dependent structural effects. Systems stabilized with Tween 60, Brij S20 and Span 60 generally exhibit higher Gi′ values (Figure 9), indicating a mechanically more robust interfacial layer compared to Tween 20 (Figure 10). The frequency sweep results are therefore consistent with the trends observed in the amplitude sweep measurements.

## 4 Conclusions

We investigated how crystallization of the dispersed lipid phase upon cooling affects interfacial rheology, depending on the molecular structure of nonionic LMEs. Emulsifiers with identical fatty acid (FA) chains (C18:0) but varying headgroups (Tween 60, BrijS20, Span 60), as well as Tweens with different FA chain lengths (Tween 20: C12:0, Tween 60: C18:0), were studied. The triglyceride phase included tristearin (TS), tripalmitin (TP), and trilaurin (TL) in MCT oil.

C18:0-based emulsifiers promoted interfacial TS crystallization, forming crystalline networks with increased viscoelasticity. Span 60 led to the most stable network due to its compact interfacial layer, which favored emulsifier-emulsifier interactions at the interface and during cooling, a crystalline emulsifier layer formed. Lower coverage (Tween 60, BrijS20) resulted in looser crystalline structures and reduced stability.

The FA chain length of the emulsifier critically influenced the interfacial rheological properties. Tween 60 (C18:0) enabled cocrystallization with TS, while Tween 20 (C12:0) remained liquid during crystallization of the dispersed phase. As a result, Tween 20 acted as a structural impurity, disrupting crystalline network formation and lowering interfacial viscoelasticity. A similar effect was observed for TP. In contrast, with TL (lower melting point), cocrystallization with Tween 20 was possible, enhancing the interfacial viscoelasticity.

These results underline the importance of matching the emulsifier’s melting behavior to that of the dispersed phase: LMEs should ideally crystallize prior to or simultaneously with the oil phase to support interfacial network formation. Moreover, dispersed-phase crystallization influences interfacial rheology due to subinterfacial effects. Overall, dense interfacial packing (as seen with Span 60) and the formation of a crystalline emulsifier layer at the interface contributed to enhanced interfacial elasticity and emulsion stability.
